# High levels of third-stage larvae (L3) overwinter survival for multiple cattle gastrointestinal nematode species on western Canadian pastures as revealed by ITS2 rDNA metabarcoding

**DOI:** 10.1186/s13071-020-04337-2

**Published:** 2020-09-10

**Authors:** Tong Wang, Russell W. Avramenko, Elizabeth M. Redman, Janneke Wit, John S. Gilleard, Douglas D. Colwell

**Affiliations:** 1 Department of Comparative Biology and Experimental Medicine, Host-Parasite Interactions Program, Faculty of Veterinary Medicine, University of Calgary, Calgary, Alberta Canada; 2 Liverstock Parasitology, Agriculture and Agri-Food Canada, Lethbridge, Alberta Canada

**Keywords:** Gastrointestinal nematodes, Overwintering, Western Canada, ITS2 rDNA metabarcoding, Tracer calves

## Abstract

**Background:**

The ability of infective larvae of cattle gastrointestinal nematode (GIN) species to overwinter on pastures in northerly climatic zones with very cold dry winters is poorly understood. This is an important knowledge gap with critical implications for parasite risk assessment and control.

**Methods:**

Infective third-stage larvae (L3) were quantified in samples of fecal pats, together with adjacent grass and soil, before and after winter on three farms in southern, central and northern Alberta. Nemabiome ITS2 metabarcoding was then performed on the harvested L3 populations to determine the species composition. Finally, parasite-free tracer calves were used to investigate if the L3 surviving the winter could infect calves and develop to adult worms in spring.

**Results:**

Farm level monitoring, using solar powered weather stations, revealed that ground temperatures were consistently higher, and less variable, than the air temperatures; minimum winter air and ground temperatures were − 32.5 °C and − 24.7 °C respectively. In spite of the extremely low minimum temperatures reached, L3 were recovered from fecal pats and grass before and after winter with only a 38% and 61% overall reduction over the winter, respectively. Nemabiome ITS2 metabarcoding assay revealed that the proportion of L3 surviving the winter was high for both *Cooperia oncophora* and *Ostertagia ostertagi* although survival of the former species was statistically significantly higher than the latter. *Nematodirus helvetinaus* and *Trichostrongylus axei* could be detected after winter whereas *Haemonchus placei* L3 could not overwinter at all. Adult *C. oncophora*, *O. ostertagi* and *N. helvetianus* could be recovered from tracer calves grazing after the winter.

**Conclusions:**

The largest proportion of L3 were recovered from fecal pats suggesting this is important refuge for L3 survival. Results also show that L3 of several GIN parasite species can survive relatively efficiently on pastures even in the extreme winter conditions in western Canada. Tracer calf experiments confirmed that overwintered L3 of both *C. oncophora* and *O. ostertagi* were capable of establishing a patent infection in the following spring. These results have important implications for the epidemiology, risk of production impact and the design of effective control strategies. The work also illustrates the value of applying ITS2 nemabiome metabarcoding to environmental samples.
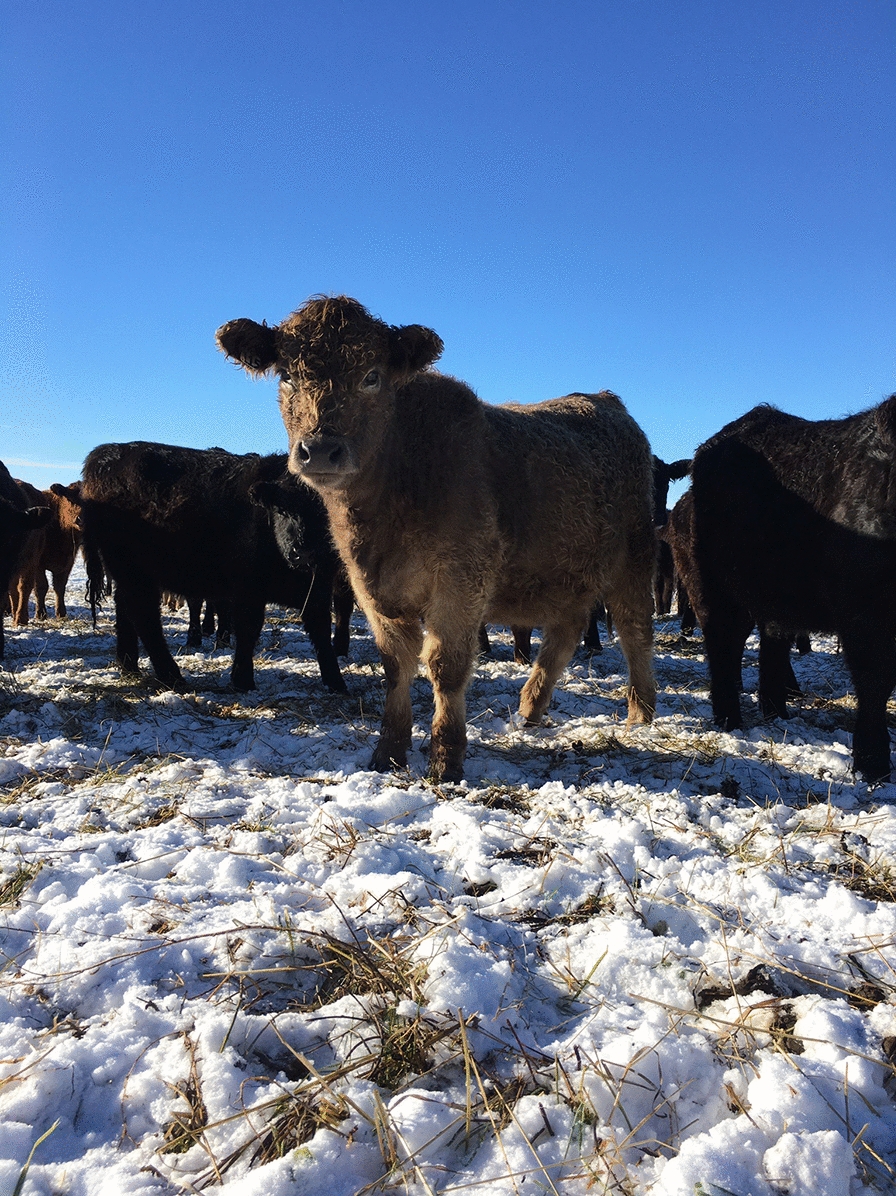

## Background

Although GIN parasites cause less clinical disease in cattle in northern latitudes than in more warm and humid climatic zones, they are still a cause of considerable production loss [1, 2]. In temperate regions, development from the egg to the infective third-stage larvae (L3) on pasture is limited to the summer grazing season because temperatures are generally too low for development of the free-living parasite stages during the winter months [3]. Consequently, pasture L3 numbers build up over the summer grazing season as they develop from eggs shed in feces of grazing cattle [4]. There are essentially two methods by which cattle GIN parasites survive northern winters; either inside the host, as adult worms and/or inhibited (hypobiotic) larvae, or in the external environment as infective L3 [5, 6]. Understanding how cattle GIN parasites survive the winter has important implications for control. For northerly regions with very cold winters, survival inside the host, particularly as inhibited larvae, is an important overwintering strategy. For example, in Canada and northern Sweden, where winters are severe, it has been described that more than 90% of GIN larvae ingested in autumn enter hypobiosis as arrested L4 stage [7]. Hypobiotic *Ostertagia* L4 larvae were predominant from November to April in Wyoming, USA, reaching a peak comprising 88% of the total nematode burden in the month of January [8]. Survival of infective L3 over the winter on pasture has been described for several major cattle and sheep GIN species, such as *Ostertagia ostertagi*, *Cooperia oncophora*, and *Teldaorsagia circumcincta* in a number of regions with cold winters including northern USA [9], Sweden [10, 11] and eastern Canada [12, 13]. However, the available information for cattle parasites is based on only a few studies conducted over 30 years ago with differing results [9, 14–16]. One study suggested that cattle GIN could not overwinter on pastures in Ontario because larvae were not found on grass until late-July, which led to the inference that this latter season L3 peak was probably from spring and early summer output of GIN eggs [15]. However, L3 were directly shown to overwinter on pasture in New Brunswick [14] and Quebec [16], with *O. ostertagi*, *C. oncophora* and *Nematodirus helvetianus* being present on pastures in the spring.

To our knowledge, there are no published data on the overwintering of cattle GIN infective L3 on pastures in cold, low humidity climates such as western Canada. This represents a major knowledge gap as it is often assumed that winter survival of cattle GIN occurs predominantly inside the host due to the low temperature extremes and low humidity that occurs during the winter months. Consequently, parasite control practices in these regions generally do not consider the role overwintered larvae in the parasite epidemiology. In this study, we investigated the ability of cattle GIN L3 to survive on western Canadian pastures over the winter. We applied ITS2 nemabiome metabarcoding as a novel approach to determine the species composition of envronmental infective L3 populations in order to determine the survival of the different GIN species. We also employed tracer calves to determine the infectivity of overwintered larvae and their ability to establish adult worm infections in the spring. We report high levels of overwintering survival of *O. ostertagi* and *C. oncophora* L3, and low levels of *N. helvetinaus* and *Trichostrongylus axei* L3 on pasture at three sites in Alberta but no overwintering survival of *Haemonchus placei* L3. These results suggest that overwintered larvae can play an important role in the epidemiology of cattle GIN species even in regions in northerly with low humidity and extremely cold winter temperatures. The work also introduces the application of nemabiome metabarcoding to determine relative abundance of ruminant GIN infective larvae from environmental samples.

## Methods

### Study sites

A field study was performed on naturally infected pastures on three commercial organic beef cattle farms located in southern Alberta (Farm 1), central Alberta (Farm 2) and northern Alberta (Farm 3) ranging between 49°49′N–55°17′N latitude and 113°95′W–118°79′W longitude. All three farms have not used anthelmintics to treat their herds in the past 10 years. During the grazing season, an intensive rotational grazing pattern was conducted by all farms; herds were moved every 1–7 days to a new cell of pasture that have not been grazed in the past 12 months (Table 1).

**Table 1:** Grazing patterns and stocking densities of the three study farms

Farm	Farm 1	Farm 2	Farm 3
Herd size	110 yearlings	61 yearlings and 3 bulls	75 cows and 75 calves
Pasture size (each)	25 acres	183 × 30 m (1.36 acres)	10 acres
Stocking rate	4.4 head/acre	47 head/acre	15 head/acre
Rotational frequency	Every 3–7 days	Every day	Every 1–3 days

### Meteorological data

One solar powered weather station (HOBO RX3000; Onset Corp, MA, USA) was set up in each farm, with probes to record pasture microclimate temperature (°C) and relative humidity (%) at 2 cm above ground level. Air temperature (°C), relative humidity (%) and precipitation (mm) were monitored with probes at 1.5 m above ground level. All the meteorological data was recorded every 15 min and transferred hourly to HOBOlink web-based software where the latest conditions could be checked.

### Experimental design

A single pasture was chosen on each of the three farms on which to undertake the study of overwintering survival of L3. The rotational grazing pattern of the three farms (Table 1) meant that the study pastures had been grazed at a high stocking density for a short period of time providing a tight temporal window of pasture contamination and, once the calves were moved from the pasture, no subsequent pasture contamination would occur until the next grazing season. Calves were grazed in August 2017 and fecal pats deposited on the study pastures (Farm 1 pasture: 12th Aug 2017, Farm 2 pasture: 15th Aug 2017, Farm 3 pasture: 6th Aug 2017). Following removal of the calves from the study pasture, both environmental sampling and tracer calves were subsequently used to investigate larval pasture contamination.

### Environmental sampling (October 2017 to May 2018)

Fecal samples, and adjacent grass and soil samples, were collected from the study pastures on 12th November 2018, 5th November 2017 and 29th October 2017 for Farm 1, Farm 2 and Farm 3 respectively (pre-winter sampling) and 15th May 2018, 8th May 2018 and 1st May 2018 for Farm 1, Farm 2 and Farm 3, respectively (post-winter sampling).

**Fig. 1:**
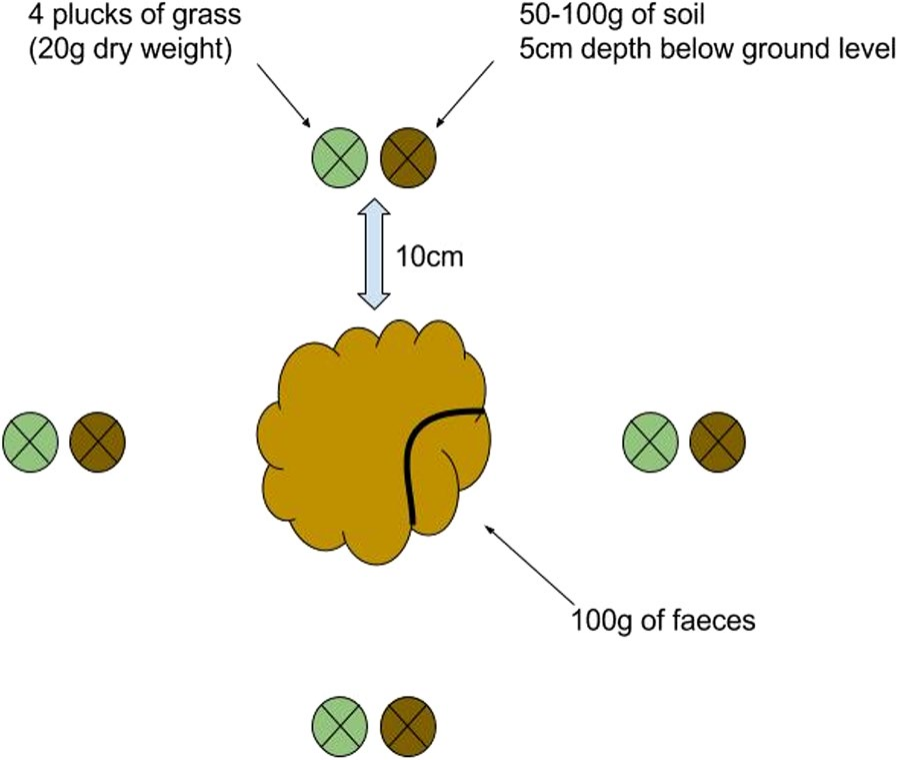
Schematic representation of environmental sampling for a fecal pat. Grass and soil samples were collected from 4 points, 10 cm away from each pat. In addition, 100 g of fecal material was collected from that pat. Samples from 4 pats were pooled to produce a single “sampling unit” and 6 such “sampling units” were collected in each farm before and after winter respectively

From each of the three pastures, environmental sampling was performed on 24 fecal pats. Environmental sampling of each fecal pat comprised the following: plucks of grass (to produce 20 g of dry weight) and 100 g of soil at a depth of 5 cm, both taken from 4 separate points 10 cm away from the edge of the fecal pat at relative positions “12, 3, 6 and 9 o’clock” (Fig. 1). It also included 100 g of fecal material collected from the edge of the fecal pat. In order to keep the laboratory work at a feasible scale, the samples from 4 fecal pats were then pooled to produce a total of 6 “sampling units” per pasture. L3 from environmental samples were enumerated and extrapolated to L3/kg dry matter.

### Tracer calves

Six-month-old Holstein steer calves, that had never been previously grazed, were purchased in March 2018 and dosed with Ivermectin using the manufacturers recommended dose (1 ml/50 kg, IVOMEC - ivermectin injection; Boehringer Ingelheim, Lyon, France) followed by a fenbendazole treatment after two weeks (5 mg/kg, Safeguard; Merck Animal Health, Kenilworth, USA). To confirm that the tracer calves were parasite-free, fecal egg counts (FEC) were performed on three occasions before turnout and found to be negative for strongyle eggs. Each pair of tracer calves was allowed to graze for three weeks. The movement of tracer calves was restricted using an electric fence to ensure they were grazing in a pre-determined section (approximately 30 × 180 m) of the pasture. After three weeks of grazing, each tracer calf pair was removed from their respective study pasture, replaced with the next pair, then taken to the Lethbridge Research and Development Centre (Lethbridge, Canada) and housed for 3 weeks in concrete pens to allow infections to reach patency. At necropsy, adult worms from the abomasum and small intestine were recovered, counted and morphologically identified [17, 18].

### L3 recovery from grass, soil and fecal samples

Grass and soil samples were processed with a floatation and sieving protocol adapted from a previous method [19]. In brief, approximately 500 g of each sample was submerged in 5 l of water with a few drops of detergent (Sunlight, Mississauga, Canada) for 3 h. The resulting water containing L3 was passed through a 200-μm sieve and then a 25-μm sieve. Fifty ml of water containing the retained L3 on the 25 μm sieve were carefully washed into a 50 ml Falcon tube using tap water followed by centrifugation at 3000× *g* for 3 min. Forty-five ml of supernatant was then removed by suction before refilling the tube with 45 ml sugar solution (specific gravity of 1.18) and centrifuged at 3000×*g* for 3 min. Supernatant containing the floated L3 was transferred into a 25-μm sieve and the sugar solution washed away using tap water. L3 were harvested from the filter by rinsing with tap water into a 15 ml tube. Finally, L3 were identified among the free-living nematodes based on the morphological indicators [20], counted using a counting chamber (Sedgewick Rafter, VWR, Radnor, USA) and the whole population was fixed in 70% ethanol for molecular analysis.

L3 from feces were recovered with a method which was modified from a coproculture protocol [21]. Approximately 50 g of fecal sample was placed in a 200 ml glass tumbler and mixed thoroughly with 50 g of vermiculite and left for 24 h. The sample was then topped up with warm tap water to the very top of the rim followed by placing an inverted plastic Petri dish on top and the whole unit was carefully inverted. The Petri dish was then filled with water and left for 4 h for L3s to swim out of the glass. Water containing L3 was finally collected into a 15 ml tube followed by enumeration of L3 and fixation in 70% ethanol for further molecular analysis.

### Adult worm recovery from tracer calves

Following slaughter, gastrointestinal contents and the organs (abomasum and small intestines) were processed separately. Briefly, abomasal or small intestinal contents were squeezed out into buckets with 5 liters 0.8% saline and allowed to settle for 2 h. Meanwhile, the organs (without content) were submerged into a separate bucket containing 0.8% saline and incubated in 37 °C for 2 h to allow worms to be released into the saline. Different buckets were used for abomasum and small intestine. After removal of organs, the saline was allowed to settle for 2 h then 2 liters of sedimented liquid, containing the worms, was collected and worms in a 200 ml aliquot were picked on a flat white tray. All harvested adult worms were fixed into 70% ethanol for morphological classification and enumeration.

### Genomic DNA preparation and ITS2 rDNA nemabiome metabarcoding

A commercial extraction kit (DNeasy PowerSoil; Qiagen, Hilden, Germany) was used to extract DNA from L3 recovered from fecal and grass environmental samples after they had been pooled by pasture. Each pool contained a range of 80–1785 strongyle L3 (details of numbers of larvae in each sample are given in Additional file 1: Tables S1 and S2). This kit was chosen mainly because of its bead-beating step (using a bead-beater) to break up the particles in these environmental samples. The protocol supplied by the manufacturer was followed and all the extractions were done in one batch on one day. Nemabiome metabarcoding was performed to determine the species composition in each sample as previously described [22]. In brief, a 350-bp fragment encompassing the rDNA ITS2 was PCR-amplified from a 1:10 dilution of genomic DNA template. A second round limited cycle PCR was performed to add combinatorial barcoding adaptors to allow amplicons from many populations to be pooled and then sequenced on an Illumina MiSeq Sequencer using a 500-cycle pair-end reagent kit (MiSeq Reagent Kits v2, MS-103-2003; Illumina, San Diego, USA). An average read depth of 23,660 was generated and samples were removed if they did not have at least 2000 reads. Details of the number of L3 and read counts (pre-quality filtering reads, post-quality filtering reads and reads assigned to reference sequences in the database) in each sample can be found in Additional file 1: Table S1 and Table S2 for pre- and post-winter results, respectively). The reads lost between steps 2 (post-quality filtering) and 3 (reads assigned to reference sequences in the database) have been checked by BLAST and they did not appear to be trichotrongylid nematodes. In order to test the reproducibility of the assay, each genomic DNA sample was PCR amplified and sequenced 4–8 independent times. Further details of protocols are available at https://www.nemabiome.ca/.

### Bioinformatics analysis

Sequence data was analysed using a bioinformatics pipeline based on Mothur version 1.36.1 [23] as previously described [24, 25]. In brief, raw paired-end reads were assembled into single contigs and then filtered to removed contigs < 200 bp or > 450 bp or those with ambiguities between the overlapping paired-end reads. A database of rDNA ITS2 reference sequences from all relevant nematodes was created from the public databases and our own reference samples (Additional file 2: Alignment S1). Reads were aligned to this database and discarded if they did not align to at least 10% of any ITS2 amplicon with at least 90% sequence similarity. The remaining sequences were classified as corresponding to reference sequence in the database with the k-nearest-neighbor method (k = 3). Sequences were classified to a higher taxonomic level if the three nearest matches did not map to a single species. Detailed information regarding this protocol can be found at www.nemabiome.ca.

### Statistical analysis

The daily mean temperature and precipitation were calculated from the meteorological data (recorded every 15 min) following download from the weather station.

L3 counts were log-transformed and analysed in a linear model, followed by Tukey’s *post-hoc* test applied to significant outcomes. The effect variables were ‘Compartment’ (grass and feces), ‘Season’ (before winter and after winter) and ‘Farm’ (Farm 1, 2 and 3). Soil L3 was excluded in the test because of the very small number of L3 collected from this compartment. Shapiro-Wilk test was used to test the normality of the dependent variable [26]. We selected the best model from the set of candidate models using Akaike’s information criterion with second-order adjustment to correct for small sample bias (AICc); the model with the lowest AICc value was chosen as the best model in the analysis [27]. The above statistical approaches were carried out in RStudio (RStudio Team, 2016, Integrated Development for R. RStudio, Inc., Boston, MA, USA; URL http://www.rstudio.com/.). We used the ‘dredge’ function in the MuMIn package to automatically select factors that provide the model with the lowest AICc value.

The alpha diversity of the ITS2 rDNA metabarcoding data for each sample was calculated using the inverse Simpson index [28] to estimate the richness and evenness of the species distribution. Beta-diversity calculations of each species between two populations (before and after winter) were calculated using the weighted Bray-Curtis dissimilarity index [29]. The method in this paper used 1000 permutation and default parameters [30] to assess how two communities differ in their species composition using a modified non-parametric t-test to assign a *P*-value of significance.

## Results

### Comparison of air and ground temperature variation over the 2017/2018 winter

**Fig. 2:**
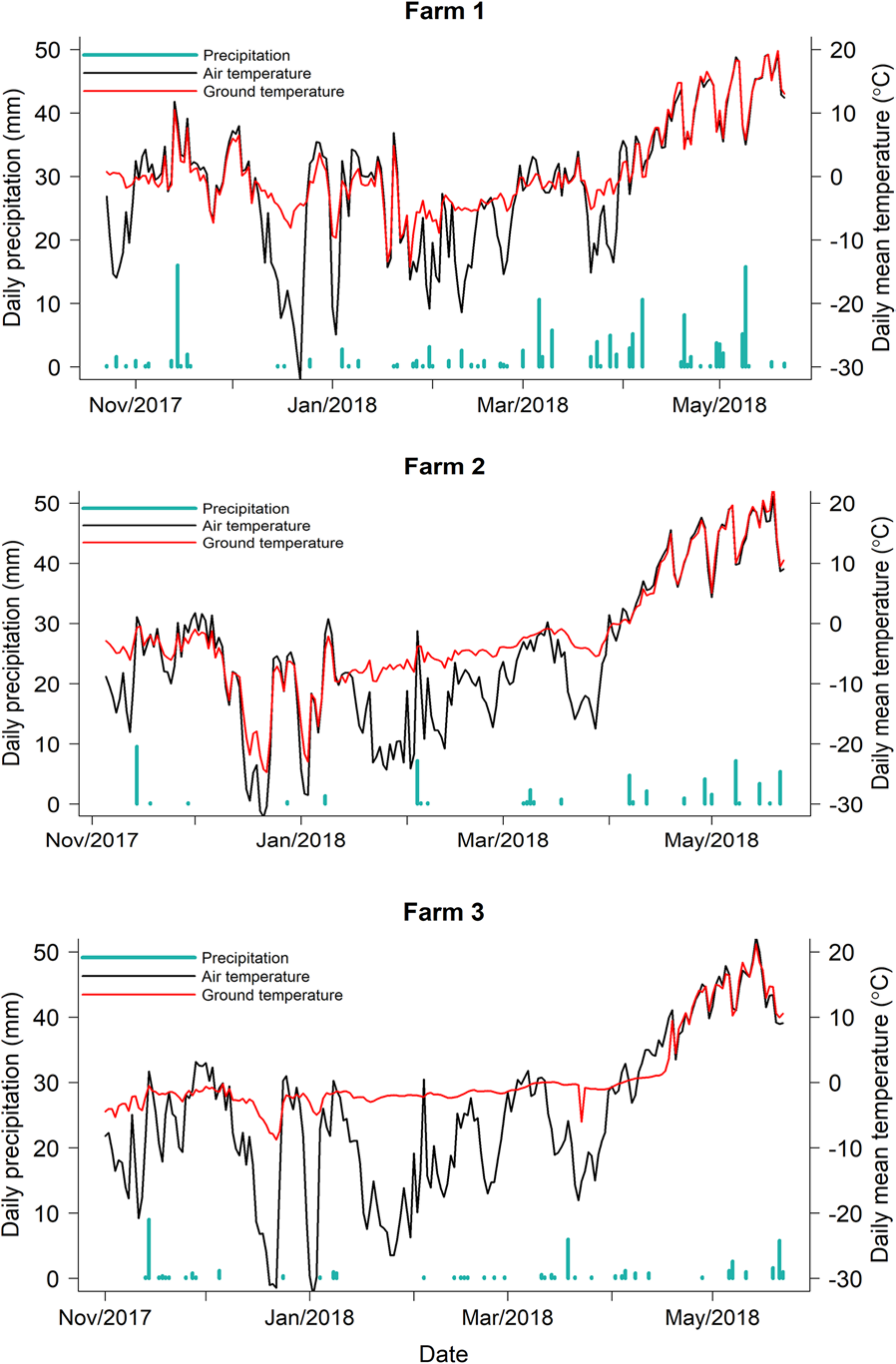
Daily mean air temperature, ground temperature and total precipitation recorded by the RX3000 Weather Station placed on each of 3 organic producers in Alberta, Canada between 10th November 2017 and 10th June 2018

The daily average air and ground temperatures were calculated from data recorded by a weather station on each farm every 15 min (Fig. 2) and the overall mean values over the entire period from 10th November 2017 to the 10th June 2018 was also determined (Table 2). On each farm, the ground temperatures were consistently higher and had less variance than the air temperature. The mean air and ground temperatures were lowest in Farm 2 and highest in Farm 1. Daily precipitation was also measured with most occurring after March (Fig. 2).

**Table 2:** Mean and minimum air and ground temperatures for the three farms. These were calculated from the data from the RX3000 Weather Stations placed on each farm between 29th October 2017 and 15th May 2018

	Farm 1	Farm 2	Farm 3
Air mean temperature (°C)	− 1.44	− 5.34	− 5.12
Air minimum temperature (°C)	− 32.09	− 32.15	− 32.5
Ground mean temperature (°C)	0.94	− 1.99	0.75
Ground minimum temperature (°C)	− 14.27	− 24.74	− 8.77

### Investigating the overwinter survival of L3 on pastures by environmental sampling and ITS2 rDNA metabarcoding

**Fig. 3:**
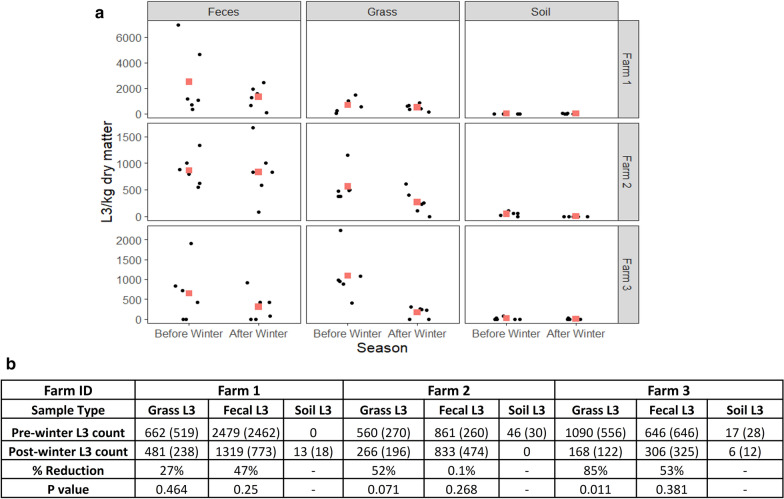
The fecal, grass and soil L3 larval counts (/kg of dry matter) in samples taken before and after winter on the three study farms. **a** Each black dot represents the L3 count of one sampling unit (each comprising individual samples pooled from 4 fecal pats as described in Methods). Each red square shows the mean of the data for the L3 counts of the 6 sampling units on each pasture. Note the different scale of y-axis. **b** Tabulated mean grass, fecal and L3 counts (/kg dry matter) in each farm pre- and post-winter with the standard deviation shown in parentheses. *P*-values show the results of the t-test on log transformed L3 counts pre- and post-winter

Sampling of fecal pats together with adjacent grass and soil was undertaken before and after winter on the three farms. Mean grass and fecal L3 counts were expressed as per kg of dry matter (DM). Overall, mean L3 counts on grass across all farms dropped from 770 L3/kg DM for the grass samples collected before winter to 304 L3/kg DM for those collected after winter, giving an overall mean reduction of 61% (Fig. 3). The mean fecal L3 count across all three farms dropped from 1329 L3/kg DM for the samples collected before winter to 820 L3/kg DM for those collected after winter, giving an overall mean reduction of 38% inside feces over the 2017/2018 winter (Fig. 3). Negligible L3 were recovered from soil both before and after winter with overall means across the three farms of 20 and 6 L3 respectively. Data for the individual sampling units and mean grass and fecal L3 count (/kg dry matter) in each farm pre- and post-winter are shown in Fig 3.

A linear model showed that all three factors, Season (*F*_(1, 70)_ = 7.81, *P* = 0.007), Compartment (*F*_(1, 70)_ = 4.17, *P* = 0.045) and Farm location (*F*_(2, 69)_ = 4.26, *P* = 0.018), had a significant effect on L3 recovery. *Post-hoc* Tukey’s HSD tests further showed that significantly more L3 (total from all three compartments) were recovered from Farm 1 than Farms 2 and 3 (*P* < 0.05)

**Fig. 4:**
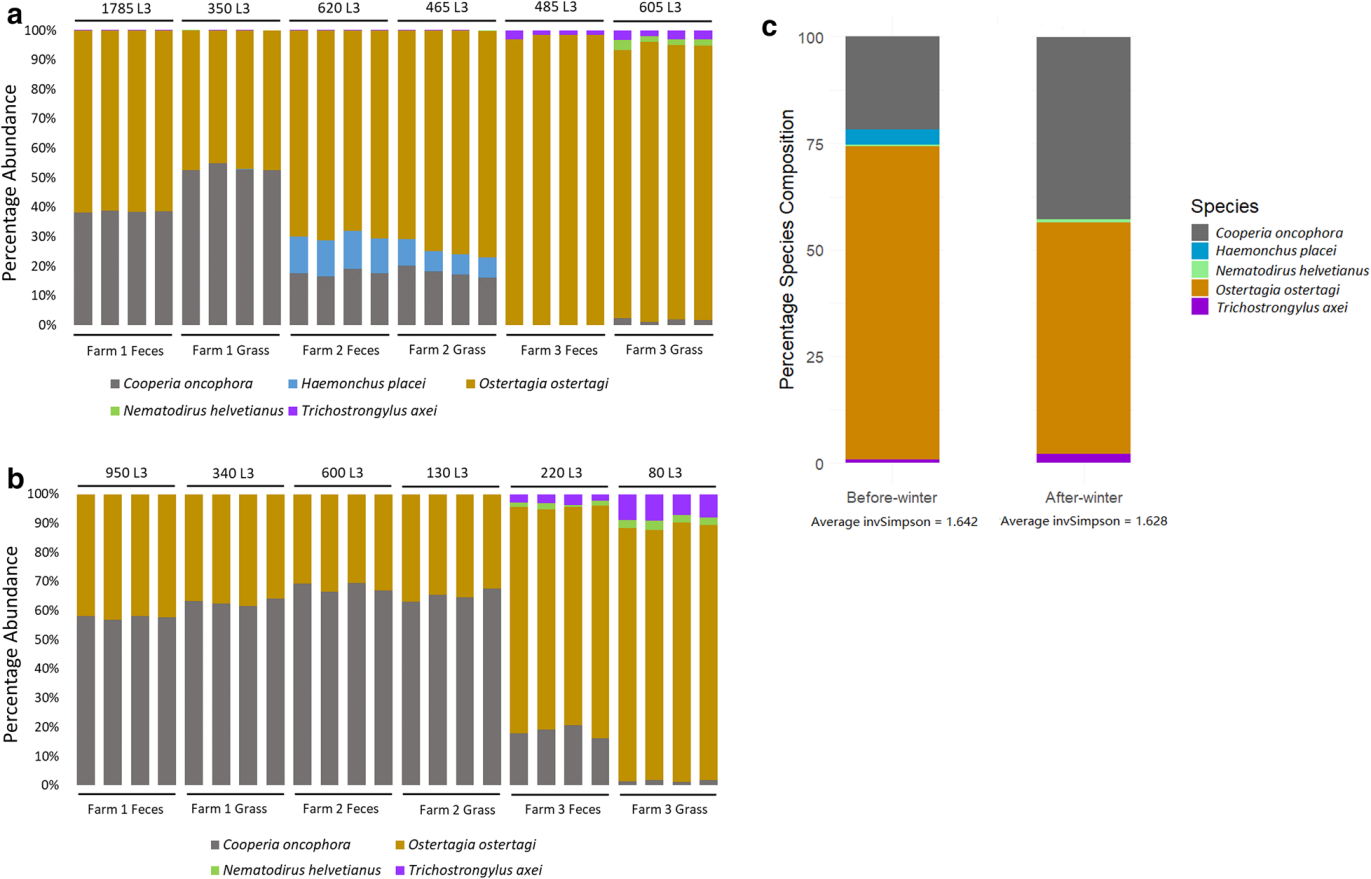
Relative species abundance in samples taken before (**a**) and after (**b**) winter as determined by ITS2 rDNA nemabiome metabarcoding applied to genomic DNA made from L3 pooled from all 6 sampling units for each farm. The number of L3 in each pool from which genomic DNA was isolated is shown above the top line and the bars within the range of each line represents the sequencing data from a separate replicate amplicon for that sample. **c** The overall mean relative abundance (%) of different parasite species present before and after winter based on the data pooled from all three farms. The overall species diversity was measured by the inverse Simpson index which showed no significant difference between samples collected before and after winter (*P* = 0.856, one-way ANOVA)

The L3 harvested before and after the winter from feces and grass were pooled by farm and fixed in ethanol. The relative abundance of different GIN species in each of these pooled larval samples was determined by ITS2 rDNA nemabiome metabarcoding. ITS2 rDNA amplicons from each set of larvae pooled by farm were independently PCR amplified and sequenced between 4–8 times. There was a high degree of reproducibility between replicates with the proportion of any one species varying between replicates of up to a maximum of 5% (Fig. 4).

The relative species abundance on each farm for the samples taken pre-winter and post-winter, and the statistical analysis of the differences between them, are shown in Fig. 4 and Tables 3, 4 and 5. The most abundant species on all three farms before the winter was *O. ostertagi* with *C. oncophora* being the second most abundant species on Farms 1 and 2 but not Farm 3 (Fig. 4a). *Haemonchus placei* was detected before the winter on Farm 2 only (Table 4), where it was the third most abundant species (range of 6–13%). *Nematodirus helvetinaus* and *T. axei* were detected in the samples taken before winter only on Farm 3 and only at low proportions (0.4–3.5%) (Fig. 4a, Table 5). For the samples taken after the winter there was a statistically significantly higher proportion of *C. oncophora* and lower proportion of *O. ostertagi* as compared to the pre-winter samples (except for the Farm 3 grass samples) (Fig. 4b). *Haemonchus placei* was not detected in any fecal or grass samples taken after the winter. *Nematodirus helvetinaus* and *T. axei* could be detected after winter at low proportions (0.8–9.1%). The overall alpha inverse Simpson index showed that overall species diversity did not change significantly between the two seasons (before winter: 1.642; after winter: 1.628; *P* = 0.856) (Fig. 4c).

**Table 3:** The mean percentage (± SE) of observed GIN species for Farm 1 in the samples before and after winter. The last column shows the beta-diversity (MetaStats) significance for differences in the individual parasite species relative abundance before and after winter

Species	Pre-winter	Post-winter	*P*-value (pre-post)
*Cooperia oncophora*	46.68 ± 7.47	60.12 ± 2.6	0.001
*Haemonchus placei*	na	na	na
*Ostertagia ostertagi*	53.10 ± 7.41	39.60 ± 2.67	0.07
*Nematodirus helvetianus*	na	na	na
*Trichostrongylus axei*	na	na	na

*Abbreviation*: na, no such species found

**Table 4:** The mean percentage (± SE) of observed GIN species for Farm 2 in the samples before and after winter. The last column shows the beta-diversity (MetaStats) significance for differences in the individual parasite species relative abundance before and after winter

Species	Pre-winter	Post-winter	*P*-value (pre-post)
*Cooperia oncophora*	18.12 ± 1.10	66.36 ± 2.02	0.002
*Haemonchus placei*	9.36 ± 2.36	0.00 ± 0.00	0.001
*Ostertagia ostertagi*	72.47 ± 2.79	33.50 ± 1.96	0.001
*Nematodirus helvetianus*	na	na	na
*Trichostrongylus axei*	na	na	na

*Abbreviation*: na, no such species found

**Table 5:** The mean percentage (± SE) of observed GIN species for Farm 3 in the samples before and after winter. The last column shows the beta-diversity (MetaStats) significance for differences in the individual parasite species relative abundance before and after winter

Species	Pre-winter	Post-winter	*P*-value (pre-post)
*Cooperia oncophora*	1.12 ± 1.01	10.62 ± 8.43	0.006
*Haemonchus placei*	na	na	na
*Ostertagia ostertagi*	95.25 ± 2.97	82.04 ± 5.33	0.004
*Nematodirus helvetianus*	1.28 ± 1.23	2.02 ± 0.68	0.001
*Trichostrongylus axei*	2.38 ± 0.89	5.31 ± 2.70	0.069

*Abbreviation*: na, no such species found

### Investigating the infectivity of overwintered L3 using tracer calves

**Fig. 5:**
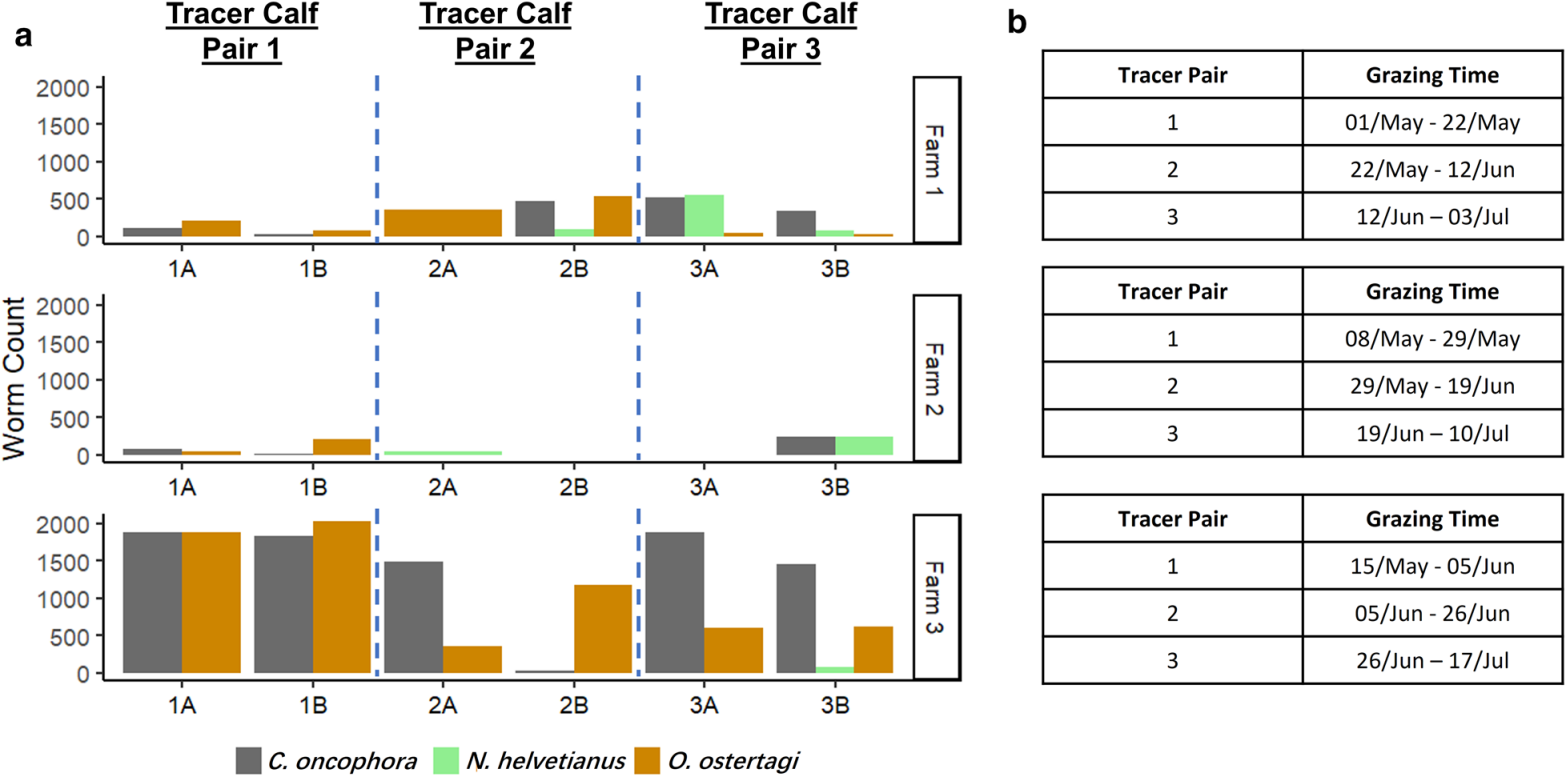
Adult worm enumeration and morphological identification for abomasal and small intestinal worms for each of tracer calves. **a** Each bar shows the total number of worms of each species from each pair of tracer calves. **b** Grazing dates for each pair of tracers in Farm 1, 2 and 3 respectively

Each farm pasture was grazed in the spring/early summer by three sets of tracer calf pairs placed on pastures at 3-weekly intervals in order to assess the infectivity of L3 surviving on pasture after the winter (Table 1). *Cooperia oncophora*, *O. ostertagi* and *N. helvetianus* were the only species for which adult worms were recovered from the tracer calves (Fig. 5). Adult worms of both *O. ostertagi* and *C. oncophora* were recovered from the first set of tracer calf pairs placed on all three farms whilst no adult worms of *N. helvetianus* were recovered. For Farm 1, adult worms of *C. oncophora*, *O. ostertagi* and *N. helvetianus* were all recovered from the second and third set of tracer calves (Fig. 5). For Farm 2, only *N. helvetianus* was recovered from the second tracer calf pair and both *C. oncophora* and *N. helvetianus* from the third set of tracer calves. For Farm 3, adult worms of *O. ostertagi* and *C. oncophora* were recovered from the first set of tracer calf pairs and all three species from the third set of tracer calves (Fig. 5).

## Discussion

### High level of L3 overwinter survival on pastures and importance of the fecal pat as a refuge

The extent to which GIN infective larvae can survive on pastures over the winter has major implications for both disease risk and control strategies [3]. In northern semi-arid climatic zones such as western Canada, prolonged periods of low, often below freezing, winter temperatures combined with low humidity are considered to be a major limiting factor for L3 survival on pastures [4]. However, there are limited empirical data on the extent to which L3 of the different GIN cattle species survive the winters in such regions most of which come from studies from over 30 years ago. This represents a major knowledge gap. GIN epidemiology can change over time due to changes in climate [31], animal movement, grazing management and the emergence of anthelmintic resistance [24].

In this study, we investigated the ability of infective L3 of different bovine GIN species to overwinter on pastures at three study sites in western Canada by harvesting of L3 from environmental samples and using ITS2 rDNA nemabiome metabarcoding to provide relative quantitation of the species present. Sampling of three compartments, grass, feces and soil, that could potentially be L3 habitat, was performed before and after winter on the three farms. Some previous studies have indicated that soil was an important reservoir for GIN L3 [32, 33]. However, we recovered very few L3 from soil both before and after winter suggesting this is not an important compartment for L3 survival at these locations. This might be related to the low moisture content or composition of the soil in the region not being favorable for L3 survival. In contrast, L3 were recovered from fecal pats and grass both before and after winter. There was only a 38% and 61% overall reduction, across the three farms, in the numbers of L3 recovered after winter, compared to before, for fecal pats and grass samples, respectively. This is a remarkably high proportion of L3 surviving over the winter on pastures. Previous studies comparing the proportion of cattle GIN species surviving the winter on pasture in cold northerly regions (Sweden, Maine USA and Quebec) have reported between 1% and 40% for *C. oncophora* and 1% to 6% for *O. ostertagi* [9, 10, 16, 34]. However, only L3 in grass and adult worms in tracer calves were used as sampling methods in these studies and fecal L3 were not examined. The high rate of L3 survival observed in our western Canadian study was in spite of the mean ambient winter temperatures being as low as − 1.44 °C, − 5.34 °C and − − 5.12 °C with minimum temperatures of − 32.09 °C, − 32.15 °C and − 32.5 °C at the three locations respectively (Fig. 2, Table 2). However, the ground temperatures, taken by probes from weather stations on each farm, were much higher and less variable over the winter with mean temperatures of 0.94 °C, − 1.99 °C and 0.75 °C with minimum temperatures of − 14.27 °C, − 24.74 °C and − 8.77 °C on Farm 1, 2 and 3, respectively (Fig. 2, Table 2). This is likely to be due to the presence of snow coverage throughout much of the winter which provides insulation from fluctuating air temperatures as well as providing a microenvironment with higher humidity than suggested by atmospheric humidity values.

The high proportion of L3 recovered from fecal pats both before and after the winter demonstrates a high level of survival of L3 inside the fecal pat. The summer of 2017 was extremely dry [35] and this may have led to a high level of retention of L3 within fecal pats exacerbating this effect. The microenviroment inside the fecal pat could potentially provide additional protection to L3 to increase the proportion that survived the winter inside the pat. The breakdown of the pat occurring after spring rainfall would then potentially release large numbers of L3 onto the pasture. There was a lower, but still high, number of L3 recovered from grass post-winter relative to pre-winter. Although this could be due to larvae overwintering directly on grass, we hypothesize it is more likely due to larvae emerging out of fecal pats in the spring when the hard crust was softened by melting snow water and rainfall promoting L3 migration out of the pat onto pasture. The importance of the fecal pat as a refuge over the winter is also consistent with the observation that the percentage survival of L3 over the winter was lower for Farm 3 (31% overall) than for Farm 1 (63% overall) and Farm 2 (74% overall). In the case of Farm 3, the ratio of L3 in the fecal pat relative to on grass in the fall was much lower (0.59:1) than for Farms 1 (3.74:1) and 2 (1.54:1). This suggests that a greater proportion of larvae had left the fecal pats and migrated onto grass in the fall for Farm 3 which could account for the subsequent lower winter survival compared to the other two farms. The importance of the fecal pat as a refuge for winter survival of infective L3 on pastures in regions with very cold low humidity winters is an important observation with practical implications for control. Removal or physical disruption of fecal pats from pastures in the fall could be an effective method to reduce overwinter survival and so be a management practice to help reduce the need for anthelmintic treatments.

### ITS2 nemabiome metabarcoding provides information on the ability of infective L3 of different cattle GIN species to overwinter on western Canadian pastures

A novel aspect of this study was the use of ITS2 nembiome metabarcoding of infective larvae recovered from environmental samples (fecal pats and grass) to determine the species composition of the trichostrongylid L3 communities. To our knowledge, this is the first time that nemabiome metabarcoding has been applied to assay populaitons of GIN infective L3 from environmental samples and it proved to be highly effective. The ITS2 nemabiome metabarcoding data indicated a statistically significant increase in the proportion of *C. oncophora* (21.8% to 42.7% of all trichostrongylid species present) and a statistically significant decrease in the proportion of *O. ostertagi* (73.5% to 54.3% of all trichostornglylid species present) between pre- and post-winter samples overall (Fig. 4, Tables 3, 4 and 5). Hence, although the proportion of L3 surviving the winter was relatively high for both species, *C. oncophora* survived the winter better than *O. ostertagi*. The proportion of *T. axei* and *N. helvetianus* was also higher in the samples taken after the winter relative to before. In contrast, *H. placei* was only present in the pre-winter fecal and grass samples of Farm 2 but was not detected at all in the post-winter samples. This suggests that L3 of this species does not survive the winter on western Canadian pastures. This is consistent with this species being better adapted to warmer climates, as is the case for *H. controtus*, which has also been shown not to overwinter on pastures in eastern Canada and Sweden [13, 36].

### Tracer calf studies confirm the infectivity of overwintered L3

Parasite-free tracer calves were used to investigate if the L3 surviving the winter could still infect calves and develop to adult worms in spring. These tracer calves had never grazed on any pastures since birth and were treated with fenbendazole before turnout. This means that any GIN recovered from their gastrointestinal tract following grazing in the spring were derived from L3 that had overwintered on pasture. The results overall showed that at least some of the overwintered L3 are capable of establishing infection in spring even though they were exposed to extreme cold conditions. *Cooperia oncophora* and *O. ostertagi* were the main species with adult worms recovered from the tracer calves in the spring consistent with the nemabiome data from environmental samples. Similarly, the absence of any *H. placei* in any of the tracer calves is also consistent with the nemabiome data from the environmental samples in suggesting this species does not overwinter effectively on western Canadian pastures.

There were some discrepancies between the nemabiome data on environmental L3 and tracer calf data for *N. helvetianus*. This species was only detected on the Farm 3 pasture by the nemabiome metabarcoding and not on the Farm 1 and 2 pastures. This result is consistent with expectations because the Farm 3 pasture was grazed by younger calves (cow/calf herd) whereas those of Farms 1 and 2 were not (yearlings) and *N. helvetianus* is generally only seen in younger calves. However, *N. helvetianus* was surprisingly present in at least some tracer animals from the pastures from all three farms albeit it at low levels and very variably. The reason for this is not clear although that could be due to a number of experimental reasons such as inconsistent recovery of *N. helvetianus* L3 from fecal pats due to variable hatching or biases in parasite establishment in the small number of individual tracer calves used.

## Conclusions

The demonstration of relatively efficient overwintering of both *O. ostertagi* and *C. oncophora* on western Canadian pastures has important implications for control. Even relatively small numbers of overwintered L3 establishing infections on grazing cattle in the spring/early summer can lead to patent infections and result in significant pasture contamination later in the grazing season. This should be considered when assessing the risk of production impacts and the potential benefits for spring early/summer anthelmintic treatments to reduce pasture contamination later in the season. Furthermore, the importance of the fecal pat as a refuge for infective L3 during the winter suggests disrupting/removing fecal pats in the fall could have significant benefits for parasite control.

## Supplementary information

**Additional file 1: Table S1.** Pre-winter sequencing results. **Table S2.** Post-winter sequencing results. Details of the number of L3 and read counts (pre-quality filtering reads, post-quality filtering reads and reads assigned to reference sequences in database) in pooled environmental samples.

**Additional file 2: Alignment S2.** Database used in the bioinformatics analysis which contains all available ITS2 rDNA sequences from the relevant nematodes. It is a combination of our own reference sequences and those from public databases.

## Data Availability

Data supporting the conclusions of this article are included in this published article and its additional files. All the raw sequences were submitted to the NCBI database (https://www.ncbi.nlm.nih.gov/Traces/study/?acc=PRJNA647918&o=acc_s%3Aa) with accession number PRJNA647918.

## Abbreviations

GIN: gastrointestinal nematode
L3: infective third-stage larvae
ITS: internal transcribed spacer
rDNA: ribosomal DNA
PCR: polymerase chain reaction
AIC: Akaike’s information criterion
